# Proposed Diagnostic Criteria and Classification of Canine Mast Cell Neoplasms: A Consensus Proposal

**DOI:** 10.3389/fvets.2021.755258

**Published:** 2021-12-10

**Authors:** Michael Willmann, Vilma Yuzbasiyan-Gurkan, Laura Marconato, Mauro Dacasto, Emir Hadzijusufovic, Olivier Hermine, Irina Sadovnik, Susanne Gamperl, Mathias Schneeweiss-Gleixner, Karoline V. Gleixner, Thomas Böhm, Barbara Peter, Gregor Eisenwort, Richard Moriggl, Zhixiong Li, Mohamad Jawhar, Karl Sotlar, Erika Jensen-Jarolim, Veronika Sexl, Hans-Peter Horny, Stephen J. Galli, Michel Arock, David M. Vail, Matti Kiupel, Peter Valent

**Affiliations:** ^1^Department/Hospital for Companion Animals and Horses, Clinic for Internal Medicine and Infectious Diseases, University of Veterinary Medicine Vienna, Vienna, Austria; ^2^Ludwig Boltzmann Institute for Hematology and Oncology, Medical University of Vienna, Vienna, Austria; ^3^Comparative Medicine and Integrative Biology Program, College of Veterinary Medicine, Michigan State University, East Lansing, MI, United States; ^4^Department of Veterinary Medical Science, University of Bologna, Ozzano dell'Emilia, Italy; ^5^Department of Comparative Biomedicine and Food Science, University of Padua, Padua, Italy; ^6^Division of Hematology and Hemostaseology, Department of Medicine I, Medical University of Vienna, Vienna, Austria; ^7^Department of Hematology, Imagine Institute Université de Paris, INSERM U1163, CEREMAST, Necker Hospital, Paris, France; ^8^Department of Clinical Pharmacology, Medical University of Vienna, Vienna, Austria; ^9^Institute of Animal Breeding and Genetics, University of Veterinary Medicine, Vienna, Austria; ^10^Department of Hematology, Hemostasis, Oncology, and Stem Cell Transplantation, Hanover Medical School, Hanover, Germany; ^11^Department of Hematology and Oncology, University Hospital Mannheim, Heidelberg University, Mannheim, Germany; ^12^Institute of Pathology, Paracelsus Medical University of Salzburg, Salzburg, Austria; ^13^Center of Pathophysiology, Infectiology and Immunology, Institute of Pathophysiology and Allergy Research, Medical University of Vienna, Vienna, Austria; ^14^The Interuniversity Messerli Research Institute, University of Veterinary Medicine Vienna, Medical University Vienna, University of Vienna, Vienna, Austria; ^15^Institute of Pharmacology and Toxicology, University of Veterinary Medicine Vienna, Vienna, Austria; ^16^Institute of Pathology, Ludwig-Maximilians University, Munich, Germany; ^17^Department of Pathology, Stanford University School of Medicine, Stanford, CA, United States; ^18^Department of Microbiology and Immunology, Stanford University School of Medicine, Stanford, CA, United States; ^19^Laboratory of Hematology, Pitié-Salpêtrière Hospital, Paris, France; ^20^Department of Medical Sciences, School of Veterinary Medicine, University of Wisconsin-Madison, Madison, WI, United States; ^21^Carbone Cancer Center, University of Wisconsin-Madison, Madison, WI, United States; ^22^Pathobiology and Diagnostic Investigation, College of Veterinary Medicine, Michigan State University, East Lansing, MI, United States

**Keywords:** canine mast cell neoplasm, classification, grading, staging, *KIT* mutations, treatment algorithms, targeted therapy

## Abstract

Mast cell neoplasms are one of the most frequently diagnosed malignancies in dogs. The clinical picture, course, and prognosis vary substantially among patients, depending on the anatomic site, grade and stage of the disease. The most frequently involved organ is the skin, followed by hematopoietic organs (lymph nodes, spleen, liver, and bone marrow) and mucosal sites of the oral cavity and the gastrointestinal tract. In cutaneous mast cell tumors, several grading and staging systems have been introduced. However, no comprehensive classification and no widely accepted diagnostic criteria have been proposed to date. To address these open issues and points we organized a Working Conference on canine mast cell neoplasms in Vienna in 2019. The outcomes of this meeting are summarized in this article. The proposed classification includes cutaneous mast cell tumors and their sub-variants defined by grading- and staging results, mucosal mast cell tumors, extracutaneous/extramucosal mast cell tumors without skin involvement, and mast cell leukemia (MCL). For each of these entities, diagnostic criteria are proposed. Moreover, we have refined grading and staging criteria for mast cell neoplasms in dogs based on consensus discussion. The criteria and classification proposed in this article should greatly facilitate diagnostic evaluation and prognostication in dogs with mast cell neoplasms and should thereby support management of these patients in daily practice and the conduct of clinical trials.

## Introduction

Mast cell (MC) tumors are hematopoietic neoplasms characterized by uncontrolled proliferation and/or accumulation of neoplastic MCs in various organ systems (1–3). In dogs, cutaneous mast cell tumors (MCTs) represent a commonly diagnosed malignancy of the skin (1–4). The most frequent clinical presentation is a solitary cutaneous nodule (1–4). However, patients can also present with multiple tumors in the skin. The clinical picture and course of cutaneous MCTs vary among patients, ranging from hairless, slowly growing skin lesions to rapidly growing, often ulcerating aggressive variants, spreading to regional lymph nodes and/or visceral organs (1–5). Rarely, these patients even progress to MC leukemia (MCL). In other patients, mucosal tissue sites or other internal organs are involved without skin lesions. Depending on the organ involved, MC-derived mediators (histamine and others) may lead to clinical signs, such as pruritus, bruising, skin swelling (edema), and/or gastrointestinal symptoms. These symptoms usually support the diagnosis of a MC neoplasm, especially when a positive Darier's sign is also demonstrable (1–4). The Darier's sign is characterized by swelling, itching and/or redness of lesional skin (MCT) after stroking or scratching.

To establish the diagnosis of MCT, a cytological examination of a fine needle aspirate is usually sufficient, but the biological behavior can only be determined by additional clinical and laboratory analyses and thus assessment of the grade and stage of the disease (1–4). Canine cutaneous MCTs were first classified by Hottendorf and Nielsen in 1967 (6). This classification was utilized by Bostock et al. in 1973 to develop a grading system (Supplementary Table 1) (7). Another and more frequently used grading system was established by Patnaik et al. in 1984 (8). This grading system divides cutaneous MCT into three grades, namely MCT consisting of well-differentiated MCs as grade 1, MCT with intermediately-differentiated MCs as grade 2, and MCT with poorly-differentiated MCs as grade 3 disease (Supplementary Table 2) (8). Due to inter-observer variations in grading and the unpredictable biological behavior of grade 2 MCT (9–11), a third 2-tier grading system was proposed (Supplementary Table 3) (9). While the Kiupel grading system is now mostly used together with the Patnaik system for prognostication of cutaneous MCT in dogs, there are still open issues to be addressed. For example, around 15% of Kiupel low-grade MCTs may have a more aggressive biological behavior (9, 12).

Independent of the histopathological grading, all MCTs are also staged based on the clinical staging system of the World Health Organization (WHO) published by Owen et al. in 1980 (13). This system includes 4 stages, based on organ involvement and the spread of disease (Supplementary Table 4). However, the WHO-based staging system does not always correlate with prognosis (14). Therefore, an adjusted staging system has recently been proposed where an additional stage with disseminated/multiple cutaneous MCTs (≥3 cutaneous MCTs) without lymph node or other organ involvement is included (Supplementary Table 5) (15). Whether this adjusted staging system can support clinical assessment of MCT patients remains to be determined. Another unsolved issue for the clinician and pathologist is how to classify and stage/grade the disease when the primary tumor site involved is different from the skin (16–27). In fact, so far, the grading and staging systems proposed for MCT were primarily established for cutaneous MCTs (8, 9, 13).

In order to discuss these open issues a group of international experts (expert faculty) in mast cell disorders from the human and veterinary fields of medicine met in Vienna in May 2019 (28). Our meeting faculty discussed open questions concerning diagnostic aspects, criteria and classification. The resulting outcomes of the conference, including an updated global classification for canine MCT, are provided in the current article.

## Proposed Classification of Canine MC Neoplasms and Minimal Diagnostic Criteria

Depending on the affected organ(s), canine MCT can be divided into cutaneous mast cell tumors (cMCTs), subcutaneous mast cell tumors (scMCTs), mucosal mast cell tumors (mMCTs), extracutaneous/extramucosal mast cell tumors (eMCTs) without skin involvement, and mast cell leukemia (MCL) (Table 1). For each category, minimal diagnostic criteria and distinct sub-variants are proposed (Tables 1, 2).

**Table 1:** Proposed classification of canine mast cell neoplasms.

**Mast cell neoplasm**	**Diagnostic criteria**
**Mast cell tumor of the skin**	Histopathologically confirmed skin mast cell tumor
Localized cutaneous MCT (cMCT)	Localized in dermis (may extend into subcutis)^*^ No lymph nodes and no other extracutaneous organ involved No evidence of MCL
Regional metastatic cMCT	Regional/Sentinel lymph nodes and no other extracutaneous organs involved as determined by microscopic investigation^**^ No evidence of MCL
Distant metastatic cMCT	Regional/Sentinel lymph nodes and/or other extracutaneous organs involved as determined by microscopic investigation^***^ No evidence of MCL
Localized subcutaneous MCT (scMCT)	Localized to subcutaneous tissue only^*^ No lymph nodes and no other extracutaneous organ involved No evidence of MCL
Regional metastatic scMCT	Regional/Sentinel lymph nodes and no other extracutaneous organs involved as determined by microscopic investigation^**^ No evidence of MCL
Distant metastatic scMCT	Regional/Sentinel lymph nodes and/or other extracutaneous organs involved as determined by microscopic investigation^***^ No evidence of MCL
**Mucosal Mast cell tumor (mMCT) (oral or intestinal)**	Histopathologically confirmed mast cell tumor localized to mucosa^*^
Localized mMCT	No lymph nodes and no other extracutaneous organs involved No skin lesions and No evidence of MCL
Regional metastatic mMCT	Regional/Sentinel lymph nodes and no other extracutaneous organs involved as determined by microscopic investigation^**^ No evidence of MCL
Distant metastatic mMCT	Regional/Sentinel lymph nodes and/or other extracutaneous organs involved as determined by microscopic investigation^***^ No skin lesions and No evidence of MCL
**Extracutaneous/extramucosal Mast cell tumor (eMCT)**	Histopathologically confirmed mast cell tumor in extracutaneous/extramucosal organs
Localized eMCT	In one extracutaneous/extramucosal organ No lymph nodes and no other extracutaneous organ involved No skin lesions and No evidence of MCL
Regional metastatic eMCT	Regional/Sentinel lymph nodes and no other extracutaneous/extramucosal organs involved as determined by microscopic investigation^**^ No evidence of MCL
Distant metastatic eMCT	Regional/Sentinel lymph nodes and/or other extracutaneous/extramucosal organs also involved as determined by microscopic investigation^***^ No skin lesions and No evidence of MCL
**Mast cell leukemia (MCL)**	Bone marrow involvement documented Circulating mast cells >10% No skin lesions
Primary	No preceding mast cell neoplasm known
Secondary	Preceding mast cell neoplasm known

* *Histopathologically confimed infiltration of cutaneous (cMCT), subcutaneous (scMCT), mucosal (mMCT) tissue by neoplastic MCs*.

** *Cytological or histopathological assessment of the draining lymph node: on cytology, lymph node infiltration is calculated as the percentage of MCs out of 1,000 total intact cells and classified according to the criteria by Krick et al. (29) into the following four categories: reactive lymphoid hyperplasia, possible metastasis, probable metastasis, certain metastasis. On histopathology, lymph node involvement is evaluated based on number of infiltrating MCs and tissue architecture disruption according to the criteria by Weishaar et al. (30) into the following four categories: histopathological nodal (HN) status 0 to 3 (HN0 to HN3). In case of probable and possible metastasis further investigation should be considered*.

*** *Cytological or histopathological assessment of the investigated organ(s): on cytology, MC infiltration is considered positive when the sample contains clustered or a high numbers of well-differentiated MCs or MCs with atypical morphology according the criteria by Stefanello et al. (31). On histopathology, organ involvement is diagnosed based on infiltrating neoplastic MCs*.

**Table 2:** Proposed grading criteria for canine mast cell (MC) neoplasms (9).

	Grade of MC neoplasm*	
Variables/criteria^**^	Low grade^*^	High grade^*^
Cell morphology	<3 multinucleated cells /10 HPF	≥3 multinucleated cells /10 HPF
Nulear morphology	<3 bizarre nuclei/10 HPF	≥3 bizarre nuclei/10 HPF
Karyomegaly	<10% of neoplastic cells	≥10% of neoplastic cells
Mitotic figures	<7 MF/10 HPF	≥7 MF/10 HPF

*MF, mitotic figures; HPF, high-power fields*.

* *Mast cell neoplasms are classified as low grade or high grade based on morphological features and proliferative activity*.

** *Each of these criteria indicates a high grade*.

Skin MCTs are divided into cutaneous and subcutaneous MCTs as defined by their location in the dermis or subcutis, determined by histopathology, and absence of criteria sufficient to establish the diagnosis of another disease variant. Depending on the involvement of the draining lymph node or other extracutaneous organs, cMCT and scMCT can be divided into localized, regional metastatic, and/or distant metastatic variants (Table 1). Metastatic variants of MCTs of the skin can mimick other types of MCTs with systemic spread. Our expert group is of the opinion that these cases should still be classified in dogs as cMCT or scMCT with metastatic progression for several reasons even if contrasting with the human classification system, where human mastocytosis is classified into localized cutaneous mastocytosis (CM) and systemic mastocytosis (SM) dependening on bone marrow (BM) infiltration (32, 33). First, stage IV canine MCTs are traditionally classified as cMCT or scMCT with metastatic involvement and not as systemic disease with skin involvement as in humans (34). Second, the primary origin of disease usually remains uncertain as unlike in the human system, dogs with skin disease usually do not undergo a BM investigation unless the disease progresses. However, previous studies with extensive staging, including BM aspirates, have rarely identified concurrent visceral MCTs in dogs with cMCTs with early nodal spread (34–37). The terminal stage of metastatic cMCT or scMCT may also resemble MCL. In these cases, the diagnosis may change and the disease can either be termed MCL-*like* metastatic progression of cMCTs or scMCTs or secondary MCL (Table 1).

In most cases with mMCTs, MC infiltrates are detected in the oral cavity whereas only rare cases of intestinal mMCTs have been described in the literature (14–16, 18). Other mucosal sites include subungual and perianal mMCTs. Again, mMCTs can be diagnosed as localized or regional and/or distant metastatic diseases.

Rarely, extracutaneous/extramucosal MCTs (eMCT) may develop (18, 19, 23, 24). These MCTs can be detected in any vascularized organ, such as the lymph nodes, spleen, liver, or uterus. In very rare cases, the BM and blood are involved, leading to the clinical picture of MCL (18, 22). Both, eMCTs and MCL are extremely rare neoplasms. In MCL, a leukemic spread of immature MCs is a diagnostic finding (at least 10% circulating MCs). In all these cases, the skin is not involved or is only involved at the mucocutaneous junctions.

## Proposed Grading and Staging in cMCT and scMCT

The most frequent clinical presentation of MC neoplasms in dogs is a solitary cutaneous nodule (1–4). Patients with cMCTs present with variable clinical features and forms of skin involvement. A positive Darier's sign may be found but is not always detected.

While cMCTs are regarded as a local epidermal cancer with possible subcutaneous involvement, scMCTs develop in the subcutis with the bulk of the neoplasm in the subcutaneous tissue surrounded by an adipose layer with no epidermal involvement. In in a smaller number of cases, some mast cells are found around hair follicles or mast cells are infiltrating the underlying musculature (25–27). While most skin MCTs are easily diagnosed by cytological examination of a fine needle aspirate, differentiating cMCTs from scMCTs and grading of cMCTs requires a biopsy and subsequent histopathological examination (1–4). Recently, different cytologic grading schemes have been proposed that either over- or underdiagnose high grade MCTs (38, 39). Therefore, a histopathological analysis of lesional skin (MCT) is the current gold standard for grading (8–12). According to previous studies, a majority of scMCTs have a more benign biological behavior than typical cMCTs (Supplementary Table 8) (25–27). However, these studies also report that among scMCTs, tumors with local recurrence and/or distant metastasis may sometimes be detected (25–27). Therefore, clinicians cannot precisely predict the clinical behavior and course of the diseases by only determining the location of a MCT within the skin, namely cMCT vs. scMCT. Moreover, a number of studies have shown that different anatomical cMCT locations are associated with a particularly poor prognosis, including perineal-perianal region, head and neck, inguinal area, scrotum, digit, and axilla (2, 40, 41). Histopathologic examination is necessary to accurately differentiate cMCTs from scMCTs and to grade cMCTs (25–27). All in all, our faculty group concludes that the subvariants of skin MCTs (cutaneous vs. subcutaneous) has to be reported in each case and may be helpful in prognostication, but in order to determine the prognosis more precisely, additional prognostication, including grading of the MCT, has to be performed.

Once the diagnosis of cMCT or scMCT has been established, staging procedures may determine the spread of the disease in various organs. Clinical staging includes a complete physical examination, a complete blood count and blood chemistry, fine needle aspiration cytology of regional lymph nodes (even if normal in size), abdominal ultrasound (with or without fine-needle aspiration of liver and spleen) and thoracic radiography (8, 9, 12, 42). Determining sentinel lymph nodes (SLNs) for aspiration instead of selecting the tributary node based on anatomical location has been shown to be the preferred method. More advanced imaging, such as CT or PET/CT, while not generally applied, may substitute for radiography and ultrasonography. In a majority of dogs, cMCTs initially spread to the SLNs (stage 2), then to the spleen and liver (stage 3), and finally into other visceral organs and, in some cases, the BM (stage 4), although lung involvement is very rare (23, 33, 34, 42). In the case of major blood count abnormalities and/or visceral involvement, a BM examination, including cytology (BM smears) and histopathology is recommended (23, 33, 34, 42). Otherwise, investigation of the BM in dogs with cMCTs and scMCTs is unlikely to be clinically helpful as the vast majority of cases won't have BM infiltration, at least at first presentation. Our expert faculty is of the opinion that the histopathological grading system of Kiupel et al. is standard in the grading of MCT (Table 2) (9). However, our faculty also recommends that the proliferation markers Ki67 and AgNOR are also included to confirm the proliferative rate and thus the biological behavior, in particular the likelihood of local recurrence, of histopathologically diagnosed low grade MCT. Furthermore, determining the KIT expression pattern and the mutational status of *KIT* will also provide valuable prognostic information. Importantly, the manner and extent of KIT mutation analysis performed and the specific mutations identified should be explicitly included in all reports and manuscripts, to allow future evaluation of the prognostic significance of specific mutations.

Finally, our faculty concluded that in patients with histopathologically diagnosed low grade cMCT and scMCT, a full staging procedure, including abdominal ultrasound and thoracic radiography, is not indicated after the surgical resection of the neoplasm due to the benign biological behavior of the disease, unless organomegaly or other signs of metastatic disease are found (25–27, 43). Nonetheless, the sentinel lymph node should always be examined.

## Mucosal Mast Cell Tumors: Proposed Diagnostic Criteria and Variants

Mast cell neoplasms involving the mucous membranes are rare. Mucosal MCTs most frequently arise in the oral cavity followed by other sites in the intestinal tract (19, 20, 44–47). Subungual and perianal mMCT may also occur. Oral and perioral MCTs have been documented to exhibit a much higher risk to metastasize (>50%) than MCTs of the skin (under 10%, but increasing depending on grade) (37, 44, 48). Therefore, an extensive staging procedure is recommended, including a complete physical, blood examination, cytological examination of the SLNs (even if normal in size), abdominal ultrasound (with fine-needle aspiration of liver and spleen regardless of the sonographic appearance) and thoracic radiography (12, 31). Due to the more aggressive biological behavior of mMCTs, local spread is common (28, 44–46). As in cMCTs, histopathological and immunohistochemical examination of the primary lesion is recommended.

Histopathological grading has not been established for mMCTs. However, tumors that exhibit features of a high grade cMCT are more likely to behave aggressively (15). Concerning treatment options, those mMCTs that have histopathologic features of a high grade cMCT, a high proliferation index, an abberant KIT pattern or harbor an internal duplication mutation in exon 11 of *KIT* may require more intensive treatment, including surgery and/or irradiation for local control as well as systemic treatment with conventional chemotherapy or with KIT tyrosine kinase inhibitors (TKI) (44, 48).

## Extracutaneuos/Extramucosal Mast Cell Tumors: Criteria and Variants

Extracutaneous/extramucosal MCTs are very rare and arise from different anatomical sites. Primary eMCT tumors have been described as originating from lymph nodes, spleen, liver, muscles, lungs, and also urethral and epidural locations (22, 49–55). However, as these sites are more likely to be involved by metastatic spread of aggressive cMCTs or mMCTs, thorough staging (Table 2) of suspected eMCT patients and their tumors should be performed. There is limited information on the treatment of eMCT, therefore our faculty is of the opinion that a thorough staging of these tumors should guide the clinician in the treatment decision, similar to the documented algorithms of cMCT (56).

## Mast Cell Leukemia (MCL)

As in humans, MCL is an extremely rare neoplasm in dogs. MCL may arise as a primary malignancy or may develop from an aggressive MCT, such as high-grade cMCT or mMCT (56, 57). The clinical course in all these patients is aggressive and the prognosis is poor, with median survivals ranging from a few weeks to a few months despite therapy. Typical findings in MCL are an increase in MCs in BM and in the peripheral blood. However, circulating MCs do not always support a diagnosis of MCL, because they are also found in patients with inflammatory diseases, regenerative anemia, severe infections, and trauma (58). Therefore, our expert faculty is of the opinion that at least 10% of circulating MCs (observed in at least two independent examinations carried out at a 2-week-interval) must be detected to make a preliminary diagnosis of MCL. The diagnosis should be confirmed through a BM aspirate and/or biopsy. MCL patients may show splenomegaly, hepatomegaly, and lymphadenopathy. Circulating or BM resident MCs in MCL are usually immature, but may sometimes be well-granulated and more mature by morphological investigation. Neoplastic MCs in MCL may display one or more mutations in the *KIT* oncogene. Although treatment responses are variable and no standard therapy is available, patients with MCL should require intensive therapy, such as multiagent chemotherapy and/or therapy with TKI directed against KIT.

## Established and Novel Diagnostic Parameters and Prognostication

While histopathological examination is mandatory to determine the grade of a MCT, a number of additional prognostic markers for MCT patients have been developed in recent years (Supplementary Table 7). For example, immunohistochemical labeling with different antibodies can support the prognostication and treatment decision in MCT patients. While KIT-immunolabeling (CD117) may be helpful in identifying an undifferentiated MCT, expression of KIT is not limited to MCs and is commonly observed in other round cell neoplasms, e.g., T-cell lymphomas (59). More importantly, different KIT labeling patterns correlate with the recurrence rate and survival in cMCT patients (60, 61). Ki67 immunohistochemistry of neoplastic cells and histochemical silver staining to count argyrophilic nucleolar organizer regions (AgNORs) are two other well-established proliferation markers. Especially in combination, these proliferation markers strongly correlate with survival of canine MCT patients and local recurrence of cMCTs (Table 3) (62–65). Low-grade cMCTs with a low proliferation index determined by Ki67 and AgNORs have been shown to have a low recurrence rate despite histopathologically dirty margins (66, 67). In addition, combining mitotic count, Ki67 and MCM7 has been described to improve the prognostic power in predicting death, especially in grade II cMCT (Table 3) (68). Whether CD2 and/or CD25 surface marker expression can support the diagnosis of MCT in dogs remains uncertain. In initial studies, conflicting results were obtained with these markers and expression of CD25 has also been reported in non-neoplastic MCs in dogs (69–71). Another interesting marker aberrantly expressed in neoplastic MC is CD30 (Ki-1) (72). However, whether CD30 can serve as a diagnostic or prognostic marker in canine MC neoplasms remains to be determined. Other markers may indicate certain oncogenic pathways such as the JAK/STAT signaling pathways which is known to stimulate cell proliferation and survival in various canine and human malignancies (73). Indeed, preclinical data using JAK/STAT inhibitors have demonstrated efficacy in canine neoplastic cell lines (74). In addition, canine neoplastic MCs display phosphorylated STAT5. However, it remains unknown whether pSTAT5 can be employed as diagnostic or prognostic marker in canine patients with MCTs.

**Table 3:** Risk assessment for local recurrence and systemic spread and/or metastasis of cMCT.

	**Risk factors**	
**Variables**	**Recurrence**	**Metastasis**
Grade I cMCT (8)^*^	–	–
Grade II cMCT (8)^*^	+/–	+/–
Grad III cMCT (8)^*^	+	+
Low grade cMCT (9)^**^	–	–
High grade cMCT (9)^**^	+	+
low AgNORxKi67 (Ag67) values (67)^***^ (independent from MCT-grade)	–	–
Low grade cMCT + low AgNORxKi67 (Ag67) values (66, 67)^***^	–	–
Low grade cMCT + high Ki67/high MCM7 (68)^****^	+	+
KIT pattern (60–62) (focal or diffuse cytoplasmic KIT expression compared with peri-membrane labeling)	+	+
KIT activating mutations (76, 80, 81) (exon 11 ITD mutations, etc.)	+	+

+*Correlates significantly with the variable*.

^−^*No correlation detectable*.

* *According to the Patnaik System, cMCT are classified as grade I-III based on morphological features, invasion and proliferative activity of mast cells*.

** *According to the Kiupel System, cMCT are classified as low-grade or high grade based on morphological features and proliferative activity with stringent cutoff values*.

*** *Low Ki67 index combined with low AgNOR values (Ag67)*.

**** *High Ki67 index and/or high minichromosome maintenance protein 7 (MCM7) score combined with low grade cMCT*.

## Molecular Studies: Current Status

A number of different *KIT* mutations are detectable in dogs with MCTs (75–78). Therefore, a detailed evaluation of the *KIT* gene may be clinically helpful. Currently, a full sequencing profile of the *KIT* gene is not routinely performed in daily veterinary practice (thus far). However, screening for a limited panel of *KIT* mutations known to be clinically relevant (activating mutations) and to occur recurrently (in exons 8, 9, and 11 of *KIT*) in MCT is recommended, particularly if the use of TKI's are contemplated (79–83). Internal tandem duplication mutations in exon 11 have consistently been associated with a more aggressive behavior, while internal tandem duplication mutations in exon 8 have not been associated with a poor prognosis (Table 3) (78, 84). While clinical studies examining TKI treatment of dogs with MCT carrying *KIT* activating mutations have documented higher response rates compared to conventional chemotherapy, controversial data have been published more recently, showing that dogs with *KIT* mutations treated with the TKI toceranib had a worse outcome compared to dogs with wild-type *KIT* MCT (81–83). Recently, the Oncology-Pathology Working Group (OPWG) came to the consensus that measuring *KIT* mutations could provide important objective information for the clinician, although the presence of a *KIT* mutation has not been definitively validated as an independent prognostic factor (85). A global gene expression analysis (microarray) on a cohort of cMCT biopies identified 13 genes clearly distinguishing differentiated from undifferentiated MCTs, thus predicting outcome (86). Another group investigated gene expression profiling on MCT FFPE-biopsies and demonstrated that 19 genes displayed at least 2-fold differences in expression in aggressive/metastasizing MCT compared to benign, non-metastasizing, MCT (87). Furthermore, a unique microRNA (miRNA) expression profile has been correlated with cMCT biological behavior, and miR-9 has been associated with MCT metastasis (88). So far, next generation sequencing (NGS) studies have not been performed using larger cohorts of MCT-derived samples. However, based on data obtained in the human system, it can be expected that additional mutations (apart from mutations in *KIT*) will be detectable in malignant mast cells and may contribute to the diagnosis and prognostication of MCT in the future (89). Therefore, our faculty is of the opinion that NGS characterizations should be explored in canine MCT studies in order to identify additional diagnostic mutations similar to the situation in humans (comparative oncology). As a minimum, the complete *KIT* mutational status should be determined, and specific mutations reported in any prognostic or therapeutic study of canine MCTs.

## Lymph Node Assessment

While regional lymph node (LN) metastasis is correlated with a worse prognosis, the diagnostic approach to detect LN metastasis is still challenging (84, 90). Also, the regional LN (RLN), which is the anatomically closest LN, may not be the draining sentinel LN (SLN) (91). More recently, a number of studies examined different techniques to identify SLN, including methylene blue dye, lymphoscintigraphy, indirect computed tomography lymphangiography (ICTL) and contrast enhanced ultrasound (CEUS) (91–97). For example, Lapsley et al. demonstrated in their study with cMCT and scMCT patients using ICTL to determine SLN that the SLN differed from RLN in 25% of the cases and the histopathology of the SLN altered the treatment recommendation in 50% of the examined cases, however, this pre-post study only included 17 MCT patients (96). Another study found a correlation between the size of cMCT or scMCT and a significantly higher risk to develop LN metastasis using lymphoscintigraphy SLN mapping (97). However, SLN mapping and biopsy of these LNs requires additional diagnostic and invasive procedures that are expensive and not always available, and therefore not widely used in veterinary practice.

Krick et al. demonstrated the efficacy of a cytological approach to determine LN metastasis using fine needle aspiration (Supplementary Table 6) (29). A histopathological classification system of regional LNs has been recommended to predict the outcome of dogs with stage II MCTs (Supplementary Table 6) (30). It has been shown recently that non-palpable/normal sized LNs may be metastatic (98), and that lymphadenectomy of metastatic LNs increases both time to progression and overall survival (99).

Considering the difficulties in identifying SLNs and verifying neoplastic MC infiltration of LNs, our faculty recommends a cytological assessment of all enlarged LNs and also SLNs (independent of size) as a diagnostic approach whenever applicable, with recognition that the technical equipment required to perform these diagnostic examinations is not available in daily practice in all centers. In addition, cytological assessement should be performed in all clearly enlarged LNs, independent of their location. In cases where the cytological findings remain unclear, a histopathological assessment of the LN is recommended. Metastatic LNs should be removed and sectioned at 0.2 mm intervals for consistent reporting.

## Identification of Distant Metastases

Considering the biological behavior and possible metastatic spread of MC neoplasms, abdominal imaging (e.g., ultrasound, CT) is strongly recommended in cases with clinical or histopathologic criteria indicative of an aggressive disease; this can reveal metastasis in abdominal LNs, the spleen and/or the liver (3, 14). Abdominal imaging usually is not required in patients that present with easily excisable solitary cMCT that lack clinical presentations associated with aggressive biology. However, when the histopathologic assessment reveals a high grade MCT or LN metastasis is detected, additional imaging studies are recommended for complete staging due to the poor prognosis of stage IV MCT patients (35). There is evidence of the low sensitivity and positive predictive value of ultrasound findings for detecting MC infiltration in the spleen and liver; thus routine ultrasound-guided aspiration of the spleen and liver should be performed in all cases deemed to be at high risk for metastasis regardless of ultrasonographic appearance (31).

## BM Investigations in High Grade MCT Patients

A detailed investigation of the BM in all dogs with MCTs is unlikely to be of clinical benefit, as the vast majority of cases are presented with solitary, low to intermediate grade tumors that develop in local tissue sites and do not involve the BM compartment. However, in a minority of cases that have blood count abnormalities and/or visceral involvement, a cytological BM investigation is recommended since BM infiltration is associated with a grave prognosis and shorter survival time (34–36).

## Concluding Remarks and Future Perspectives

Canine MC neoplasms are frequent tumors with a wide range of anatomical patterns and variable clinical behavior. In most cMCTs, the diagnostic approach, prognostication, and treatment recommendations can be made by using established guidelines and algorithms. However, there are different variants of MCTs in dogs with unclear clinical behavior and prognosis, such as scMCTs, mMCTs or MC neoplasms arising in other organs. A group of experts met in a Working Conference in 2019 to address open issues in the field of canine MC neoplasms. At this conference, the group discussed, and ultimately proposed, consensus criteria for the classification of all canine MC neoplasms. These criteria and proposed classification should facilitate diagnosis and prognostication, and also treatment, in the various forms of MCT encountered in clinical practice, as well as in clinical trials.

## Author Contributions

SG, MW, PV, and EH organized the working conference and the pre- and post-conference discussion. All authors actively participated in the consensus discussion and in the working conference in Vienna, contributed to the formulation of consensus statements, the preparation of the consensus manuscript, and approved the final version of the document.

## Funding

This study was supported by the Austrian Science Fund (FWF), project P32470-B, SFB grants F4701-B20 and F4704-B20, and a Cancer Stem Cell Grant of the Medical University of Vienna, EJ-J was supported by FWF project SFB F4606-B28, and ZL was supported by the Deutsche Forschungsgemeinschaft (grant: Li 1608/5-1). This project was also supported by the research collaboration between the Vetmeduni Vienna and the Medical University Vienna working in the field of comparative oncology/medicine in der Vienna Life Sciene region.

## Conflict of Interest

PV received research grants from Celgene, Novartis, Pfizer, and Incyte. The remaining authors declare that the research was conducted in the absence of any commercial or financial relationships that could be construed as a potential conflict of interest. The reviewer BR declared a shared affiliation, though no other collaboration, with several of the authors MW, RM, and VS, to the handling editor.

## Publisher's Note

All claims expressed in this article are solely those of the authors and do not necessarily represent those of their affiliated organizations, or those of the publisher, the editors and the reviewers. Any product that may be evaluated in this article, or claim that may be made by its manufacturer, is not guaranteed or endorsed by the publisher.
