# Supplementary material for: Proposed Diagnostic Criteria and Classification of Canine Mast Cell Neoplasms: A Consensus Proposal
Source: Front Vet Sci. 2021 Dec 10;8:755258. doi: 10.3389/fvets.2021.755258 (PMC8702826; doi:10.3389/fvets.2021.755258)
Supplement: Supplementary file 1 [file Data_Sheet_1.pdf]

Supplemental Data to the Manuscript `Willmann et al`:

Proposed Diagnostic Criteria and Classification of Canine Mast Cell Neoplasms: A Consensus Proposal

Table S1

Bostock Histopathological Grading System for Canine Cutaneous MC Tumors (1973)(1)

	Tumor Grade		
	I<br>high grade	II<br>intermediate grade	III<br>low grade
Cell morphology	Highly cellular, un-differentiated cytoplasmic boundaries, irregular size and shape of nuclei, frequent mitoses, sparse cytoplasmic granules.	Cells closely packed with indistinct cytoplasmic boundaries, nucleus-to-cytoplasmic ratio lower than anaplastic, frequent mitoses, more granules than anaplastic.	Clearly defined cytoplasmic boundaries with regular, spheric, or ovoid nuclei; mitoses rare or absent; cytoplasmic granules large, deep staining, and abundant.

Table S2

Patnaik Morphologic Grading Classification for Canine Cutaneous MC Tumors (1984)(2)

	Tumor Grade		
	I	II	III
Location	Dermis and interfollicular Spaces	Infiltrate lower dermal and subcutaneous tissue; some extend to skeletal muscles or surrounding tissues	Replace subcutaneous and deep tissues
Cell morphology	Round, monomorphic, ample distinct cytoplasm with medium-sized granules	Round to ovoid, moderately pleomorphic, with scattered spindle and giant cells; most cells distinct cytoplasm with fine granules, but some with indistinct cytoplasm and large/hyperchromic granules	Round, ovoid, spindle shaped, pleomorphic, medium sized; cytoplasm indistinct with granules that are fine or not obvious; many giant cells and scattered multi-nucleated cells
Nuclear morphology	Round, condensed chromatin	Round to indented with scattered chromatin and single nucleoli; some with double nuclei	Indented to round vesiculated, with 1 or more prominent nucleoli; common bi-nucleated cells
Architecture, Cellularity, stromal Reaction	Arranged in rows or small groups, separated by mature collagen fibers of the dermis	Moderately to highly cellular; arranged in groups with thin fibrovascular stroma (some-times thick and fibro-collagenous with areas of hyalinization). Sometimes, neoplastic mast cells can infiltrate in the lower dermis or even subcutaneous tissue; some tumors may even infiltrate into the skeletal muscles or surrounding tissues	Cellular, arranged in closely packed sheets; stroma fibrovascular or thick and fibrocollagenous with areas of hyalinization
Mitotic figures	None	Rare (0-2/HPF)	Common (3-6/HPF)
Edema and necrosis	Minimal	Areas of diffuse edema and necrosis	Edema, hemorrhage, and and necrosis common

HPF, high-power field

Table S3

Kiupel Two-Tier Grading Criteria for Canine Cutaneous MC Tumors (2011)(3)

Criterion*	Tumor Grade: low grade	high grade*
Mitotic figures	< 7 MF/10 HPF	≥ 7 MF/10 HPF
Cell Morphology	< 3 multinucleated cells /10 HPF	≥ 3 multinucleated cells /10 HPF
Nuclear Morphology	< 3 bizarre nuclei/10 HPF	≥ 3 bizarre nuclei/10 HPF
Karyomegaly	< 10% of neoplastic cells	≥ 10% of neoplastic cells

*Each of these criteria is sufficient to assign a high grade. Abbreviations: MF, mitotic figures; HPF, high-power fields

Table S4

WHO staging system for canine cutaneous mast cell neoplasms (4)

Clinical Staging	Criteria
Stage I	One tumor confined to dermis without regional lymph node involvement
Stage II	One tumor confined to dermis, with regional lymph node involvement
Stage III	Multiple dermal tumors or large infiltrating tumor with or without regional lymph node involvement
Stage IV	Any tumor with distant metastasis or recurrence with metastasis

(4) Owen et al., 1980

Table S5

Proposed amendment to the WHO staging system for canine cutaneous MCTs (5)

Clinical Staging	Criteria
Stage I	Single tumor, without regional lymph node involvement
Stage II	Multiple tumors (≥3), without regional lymph node involvement
Stage III	Single tumor, with regional lymph node involvement
Stage IV	Large and infiltrative tumors, without delineation, or multiple tumors (≥3), with regional lymph node involvement
Stage V	Any tumor with distant metastasis, including bone marrow invasion and the presence of mast cells in the peripheral blood

Compared to the original WHO staging system (Table S4), Stage II presenting with multiple cutaneous tumors, was included (5).

Table S6

Clinical differences of the subvariants of cutaneous MCT

	Cutaneous MCT (3,34,35)	Subcutaneous MCT (19,36)
Recurrence rate	10.5% (3)	8% (19) – 9% (36)
Metastatic disease	18.7 (34) – 22.9% (3)	4% (19) – 5.7% (36)
MCT-associated death	11.6% (3) – 29.2% (35)	5% (36) – 9% (19)

MCT, mast cell tumor. Kiupel et al., 2011 (3); Thompson et al., 2011 (19), Stefanello et al., 2015 (34); Sabattini et al., 2015 (35); Newman et al., 2007 (36).

Table S7

Prognostic Markers for Canine Mast Cell Neoplasms

	References
Histologic Grade	Patnaik (2), Kiupel (3), Thamm (8), Webster (9), Giantin (10), Sledge (11)
Clinical stage	Krick (12), Warland (13), Worley (14), Murphy (15), Hillman (16), Horta (5)
Sentinel Lymph Node Involvement	Worley (14), Grimes (17), Fournier (18), Lapsley (19), Ferrari (20)
Proliferation Marker	
Mitotic Count	Kiupel (3), Horta (5), Thompson (21), Romansik (22), Thompson (23), Bertram (24), Vascellari (25), Berlato (26)
Ki67	Horta (5), Webster (9), Thompson (21), Vascellari (25), Berlato (26), Scase (27), Abadie (28), Seguin (29), Smith (30)
AgNOR	Thamm (8), Webster (9), Scase (27), Webster (31)
IHC KIT pattern	Horta (5), Giantin (10), Thompson (19), Kiupel (32), Reguera (33)
KIT Mutation	Horta (5), Giantin (10), Sledge (11), Webster (34), Takeuchi (35), London (36)
Other Variables	
Response to TKI treatment	Horta (37)

Ki67, Ki67 nuclear protein; IHC KIT pattern, immunohistochemical KIT pattern; AgNOR, Argyrophilic Nucleolar organizer regions.

Table S8

Comparison of cytological and histopathological evaluation of lymph node MCT metastasis according to Krick (6) and Weishaar (7)

Interpretation	Cytologic Criteria*	Histopathologic Criteria**	Histopathologic Classification
non-metastatic	No MCs seen	None to rare (0-3), scattered, individualized (isolated) MCs in sinuses (subcapsular, paracortical or medullary) and/or parenchyma per x400 field (0-3 MCs per x400 field), or does not meet criteria for any other classification below	HN0
Reactive lymphoid Hyperplasia	Greater than 50% small lympho-cytes with a mixed population of prolymphocytes, lymphoblasts, plasma cells, and/or few to moderate numbers of macrophages, neutrophils, and eosinophils, and/or rare individual MCs		
Possible metastasis/ pre-metastatic	On at least one slide, two to three incidences of MCs in aggregates of two to three cells	Greater than 3 individualized (isolated) MCs in sinuses (sub-capsular, paracortical or medullary) and/or parenchyma in a minimum of 4 x400 fields (unless otherwise stated at least 4 x400 fields each, which contain more than 3 MCs)	HN1
Probable metastasis/ early metastasis	On at least one slide, greater than three foci of MCs in aggregates of two to three cells and/or two to five aggregates of more than three MCs	Aggregates (clusters) of MCs (≥3 associated cells) in sinuses (sub-capsular, paracortical or medullary) and/or parenchymal, or sinusoidal sheets of MCs	HN2
Certain metastasis/ overt metastasis	On at least one slide, effacement of lymphoid tissue by MCs, and/ or the presence of aggregated, poorly differentiated MCs with pleomorphism, anisocytosis, anisokaryosis, and/or decreased or variable granulation, and/or greater than five aggregates of more than three MCs	Disruption or effacement of normal nodal architecture by discrete foci, nodules, sheets or overt masses composed of MCs	HN3

*Krick et al., 2009 (6); **Weishaar et al., 2014 (7); MCs, mast cells; HN 0-3, histological nodal (HN) status 0-3

References

1. Bostock DE. The prognosis following surgical removal of mastocytomas in dogs. J Small Anim Pract. 1973;14:27-41. DOI: 10.1111/j.1748-5827.1973.tb06891.x

2. Patnaik AK, Ehler WJ, MacEwen EG. Canine cutaneous mast cell tumor: morphologic grading and survival time in 83 dogs. *Vet Pathol.* (1984) 21:469-74. DOI: 10.1177/030098588402100503

3. Kiupel M, Webster JD, Bailey KL, Best S, DeLay J, Detrisac CJ, et al. Proposal of a 2-tier histologic grading system for canine cutaneous mast cell tumors to more accurately predict biological behavior. *Vet Pathol.* (2011) 48:147-55. DOI: 10.1177/0300985810386469

4. Owen LA. TNM Classification of Tumours in Domestic Animals. Geneva, Switzerland: *World Health Organization.* (1980) 53.

5. Horta RS, Lavalle GE, Monteiro LN, Souza MCC, Cassali GD, Araújo RB. Assessment of Canine Mast Cell Tumor Mortality Risk Based on Clinical, Histologic, Immunohistochemical, and Molecular Features. *Vet Pathol.* (2018) 55:212-23. DOI: 10.1177/0300985817747325

6. Krick EL, Billings AP, Shofer FS, Watanabe S, Sorenmo KU. Cytological lymph node evaluation in dogs with mast cell tumours: association with grade and survival. *Vet Comp Oncol.* (2009) 7:130-8. DOI: 10.1111/j.1476-5829.2009.00185.x

7. Weishaar KM, Thamm DH, Worley DR, Kamstock DA. Correlation of nodal mast cells with clinical outcome in dogs with mast cell tumour and a proposed classification system for the evaluation of node metastasis. *J Comp Pathol.* (2014) 151:329-38. DOI: 10.1016/j.jcpa.2014.07.004

8. Thamm DH, Turek MM, Vail DM. Outcome and prognostic factors following adjuvant prednisone/vinblastine chemotherapy for high-risk canine mast cell tumour: 61 cases. *J Vet Med Sci.* (2006) 68:581-7. DOI: 10.1292/jvms.68.581

9. Webster JD, Yuzbasiyan-Gurkan V, Thamm DH, Hamilton E, Kiupel M. Evaluation of prognostic markers for canine mast cell tumors treated with vinblastine and prednisone. *BMC Vet Res.* (2008) 4:32. DOI: 10.1186/1746-6148-4-32

10. Giantin M, Vascellari M, Morello EM, Capello K, Vercelli A, Granato A, et al. c-KIT messenger RNA and protein expression and mutations in canine cutaneous mast cell tumors: correlations with post-surgical prognosis. *J Vet Diagn Invest.* (2012) 24:116-26. DOI: 10.1177/1040638711425945

11. Sledge DG, Webster J, Kiupel M. Canine cutaneous mast cell tumors: A combined clinical and pathologic approach to diagnosis, prognosis, and treatment selection. *Vet J.* (2016) 215:43-54. DOI: 10.1016/j.tvjl.2016.06.003

12. Krick EL, Kiupel M, Durham AC, Thaiwong T, Brown DC, Sorenmo KU. Investigating Associations Between Proliferation Indices, C-kit, and Lymph Node Stage in Canine Mast Cell Tumors. *J Am Anim Hosp Assoc.* (2017) 53:258-64. DOI: 10.5326/JAAHA-MS-6265

13. Warland J, Amores-Fuster I, Newbury W, Brearley M, Dobson J. The utility of staging in canine mast cell tumours. *Vet Comp Oncol.* (2014) 12:287-98. DOI: 10.1111/vco.12012

14. Worley DR. Incorporation of sentinel lymph node mapping in dogs with mast cell tumours: 20 consecutive procedures. *Vet Comp Oncol.* (2014) 12:215-26. DOI: 10.1111/j.1476-5829.2012.00354.x

15. Murphy S, Sparkes AH, Blunden AS, Brearley MJ, Smith KC. Effects of stage and number of tumours on prognosis of dogs with cutaneous mast cell tumours. *Vet Rec.* (2006) 158:287-91. DOI: 10.1136/vr.158.9.287

16. Hillman LA, Garrett LD, de Lorimier LP, Charney SC, Borst LB, Fan TM. Biological behavior of oral and perioral mast cell tumors in dogs: 44 cases (1996-2006). *J Am Vet Med Assoc.* (2010) 237:936-42. DOI: 10.2460/javma.237.8.936

17. Grimes JA, Secrest SA, Wallace ML, Laver T, Schmiedt CW. Use of indirect computed tomography lymphangiography to determine metastatic status of sentinel lymph nodes in dogs with a pre-operative diagnosis of melanoma or mast cell tumour. *Vet Comp Oncol.* (2020) 18:818-824. doi: 10.1111/vco.12592.

18. Fournier Q, Thierry F, Longo M, Malbon A, Cazzini P, Bisson J, et al. Contrast-enhanced ultrasound for sentinel lymph node mapping in the routine staging of canine mast cell tumours: A feasibility study. *Vet Comp Oncol.* (2021) 19:451-462. doi: 10.1111/vco.12647.

19. Lapsley J, Hayes GM, Janvier V, Newman AW, Peters-Kennedy J, Balkman C, et al. Influence of locoregional lymph node aspiration cytology vs sentinel lymph node mapping and biopsy on disease stage assignment in dogs with integumentary mast cell tumors. *Vet Surg.* (2021) 50:133-141. doi: 10.1111/vsu.13537.

20. Ferrari R, Boracchi P, Chiti LE, Manfredi M, Giudice C, De Zani D, et al. Assessing the Risk of Nodal Metastases in Canine Integumentary Mast Cell Tumors: Is Sentinel

Lymph Node Biopsy Always Necessary? *Animals (Basel).* (2021) 11:2373. doi: 10.3390/ani11082373.

21. Thompson JJ, Pearl DL, Yager JA, Best SJ, Coomber BL, Foster RA. Canine subcutaneous mast cell tumor: characterization and prognostic indices. *Vet Pathol.* (2011) 48:156-68. DOI: 10.1177/0300985810387446

22. Romansik EM, Reilly CM, Kass PH, Moore PF, London CA. Mitotic index is predictive for survival for canine cutaneous mast cell tumors. *Vet Pathol.* (2007) 44:335-41. DOI: 10.1354/vp.44-3-335

23. Thompson JJ, Yager JA, Best SJ, Pearl DL, Coomber BL, Torres RN, et al. Canine subcutaneous mast cell tumors: cellular proliferation and KIT expression as prognostic indices. *Vet Pathol.* (2011) 48:169-81. DOI: 10.1177/0300985810390716

24. Bertram CA, Aubreville M, Gurtner C, Bartel A, Corner SM, Dettwiler M, et al. Computerized Calculation of Mitotic Count Distribution in Canine Cutaneous Mast Cell Tumor Sections: Mitotic Count Is Area Dependent. *Vet Pathol.* (2020) 57:214-26. DOI: 10.1177/0300985819890686

25. Vascellari M, Giantin M, Capello K, Carminato A, Morello EM, Vercelli A, et al. Expression of Ki67, BCL-2, and COX-2 in canine cutaneous mast cell tumors: association with grading and prognosis. *Vet Pathol.* (2013) 50:110-21. DOI: 10.1177/0300985812447829

26. Berlato D, Murphy S, Laberke S, Rasotto R. Comparison of minichromosome maintenance protein 7, Ki67 and mitotic index in the prognosis of intermediate Patnaik grade cutaneous mast cell tumours in dogs. *Vet Comp Oncol.* (2018) 16:535-43. DOI: 10.1111/vco.12412

27. Scase TJ, Edwards D, Miller J, Henley W, Smith K, Blunden A, et al. Canine mast cell tumors: correlation of apoptosis and proliferation markers with prognosis. *J Vet Intern Med.* (2006) 20:151-8. DOI: 10.1892/0891-6640(2006)20[151:cmctco]2.0.co;2

28. Abadie JJ, Amardeilh MA, Delverdier ME. Immunohistochemical detection of proliferating cell nuclear antigen and Ki-67 in mast cell tumors from dogs. *J Am Vet Med Assoc.* (1999) 215:1629-34.

29. Séguin B, Besancon MF, McCallan JL, Dewe LL, Tenwolde MC, Wong EK, et al. Recurrence rate, clinical outcome, and cellular proliferation indices as prognostic indicators after incomplete surgical excision of cutaneous grade II mast cell tumors: 28 dogs (1994-2002). *J Vet Intern Med.* (2006) 20:933-40. DOI: 10.1892/0891-6640(2006)20[933:rrcoac]2.0.co;2

30. Smith J, Kiupel M, Farrelly J, Cohen R, Olmsted G, Kirpensteijn J, et al. Recurrence rates and clinical outcome for dogs with grade II mast cell tumours with a low AgNOR count and Ki67 index treated with surgery alone. *Vet Comp Oncol.* (2017) 15:36-45. DOI: 10.1111/vco.12140

31. Webster JD, Yuzbasiyan-Gurkan V, Miller RA, Kaneene JB, Kiupel M. Cellular proliferation in canine cutaneous mast cell tumors: associations with c-KIT and its role in prognostication. *Vet Pathol.* (2007) 44:298-308. DOI: 10.1354/vp.44-3-298

32. Kiupel M, Webster JD, Kaneene JB, Miller R, Yuzbasiyan-Gurkan V. The use of KIT and tryptase expression patterns as prognostic tools for canine cutaneous mast cell tumors. *Vet Pathol.* (2004) 41:371-7. DOI: 10.1354/vp.41-4-371

33. Reguera MJ, Rabanal RM, Puigdemont A, Ferrer L. Canine mast cell tumors express stem cell factor receptor. *Am J Dermatopathol.* (2000) 22:49-54. DOI: 10.1097/00000372-200002000-00010

34. Webster JD, Yuzbasiyan-Gurkan V, Kaneene JB, Miller R, Resau JH, Kiupel M. The role of c-KIT in tumorigenesis: evaluation in canine cutaneous mast cell tumors. *Neoplasia.* (2006) 8:104-11. DOI: 10.1593/neo.05622

35. Takeuchi Y, Fujino Y, Watanabe M, Takahashi M, Nakagawa T, Takeuchi A, et al. Validation of the prognostic value of histopathological grading or c-kit mutation in canine cutaneous mast cell tumours: a retrospective cohort study. *Vet J.* (2013) 196:492-8. DOI: 10.1016/j.tvjl.2012.11.018

36. London CA, Malpas PB, Wood-Follis SL, Boucher JF, Rusk AW, Rosenberg MP, et al. Multi-center, placebo-controlled, double-blind, randomized study of oral toceranib phosphate (SU11654), a receptor tyrosine kinase inhibitor, for the treatment of dogs with recurrent (either local or distant) mast cell tumor following surgical excision. *Clin Cancer Res.* (2009) 15:3856-65. DOI: 10.1158/1078-0432.CCR-08-1860

37. Horta RDS, Giuliano A, Lavalle GE, Costa MP, de Araújo RB, Constantino-Casas F, et al. Clinical, histological, immunohistochemical and genetic factors associated with measurable response of high-risk canine mast cell tumours to tyrosine kinase inhibitors. *Oncol Lett.* (2018) 15:129-36. DOI: 10.3892/ol.2017.7323
